# The Cultivation of Pure Altruism via Gratitude: A Functional MRI Study of Change with Gratitude Practice

**DOI:** 10.3389/fnhum.2017.00599

**Published:** 2017-12-12

**Authors:** Christina M. Karns, William E. Moore, Ulrich Mayr

**Affiliations:** ^1^Department of Psychology, University of Oregon, Eugene, OR, United States; ^2^Department of Psychology, Harvard University, Cambridge, MA, United States

**Keywords:** gratitude, altruism, ventromedial prefrontal cortex, fMRI, practice

## Abstract

Gratitude is an emotion and a trait linked to well-being and better health, and welcoming benefits to oneself is instrumentally valuable. However, theoretical and empirical work highlights that gratitude is more fully understood as an intrinsically valuable moral emotion. To understand the role of neural reward systems in the association between gratitude and altruistic motivations we tested two hypotheses: First, whether self-reported propensity toward gratitude relates to fMRI-derived indicators of “pure altruism,” operationalized as the neural valuation of passive, private transfers to a charity versus to oneself. In young adult female participants, self-reported gratitude and altruism were associated with “neural pure altruism” in ventromedial prefrontal cortex (VMPFC) and nucleus accumbens. Second, whether neural pure altruism can be increased through practicing gratitude. In a double-blind study, we randomly assigned participants to either a gratitude-journal or active-neutral control journal group for 3 weeks. Relative to pre-test levels, gratitude journaling increased the neural pure altruism response in the VMPFC. We posit that as a context-dependent value-sensitive cortical region, the VMPFC supports change with gratitude practice, a change that is larger for benefits to others versus oneself.

## Introduction

Theoretical conceptions of gratitude have been reaching consensus that gratitude is distinct from appreciation or gladness, because rather than appreciating a general state of affairs, it involves a social component of recognizing the role of benefactors (Carr, 2013; Gulliford et al., 2013). Psychological characterizations of gratitude also highlight its moral nature by extending the concept beyond a positive emotional response to a benefit. Instead gratitude involves recognizing that others have acted on ones own behalf, recognizing a moral exemplar, and motivating expression of ones gratitude (McCullough et al., 2001). Furthermore, expression of gratitude involves more than direct reciprocity to a benefactor, but extends to altruistic actions toward others (DeSteno et al., 2010).

Through a small but growing body of neuroimaging evidence, there are early indications that various laboratory instantiations of gratitude involve neural responses in value-sensitive regions of the medial prefrontal cortex. However, it has not been established whether gratitude is also related to what has been described in economic terms as “pure altruism.” Pure altruism is a signature of utility or reward that cannot be attributed to impure motivations such as the warm glow from making an altruistic choice, enhanced social status through being observed by others, or potential future benefits to the self through reciprocity (Andreoni, 1990). Here, we test two related predictions about the relationship between gratitude and pure altruism using a neural measure of reward for passive and private transfers of money to charity (Harbaugh et al., 2007; Hubbard et al., 2016). Our first prediction was that if gratitude fosters an increased tendency toward pure altruism, then individuals with higher levels of trait self-reported gratitude would not only endorse altruistic values in self-report and behavioral measures, but would also show a higher degree of neural pure altruism for benefits to others versus the self (Clithero and Rangel, 2014). Our second related prediction was that if gratitude motivates altruism, practicing gratitude over a period of weeks should increase the neural pure altruism response reflecting heightened attunement toward benefits to others versus the self. This is also the first longitudinal fMRI study (with both pre and post-test neural measurements) showing change as a result of gratitude practice. Other novel features of the study include an active control group and a relatively brief 3-week journaling period.

### Gratitude Is Good for You

Although theoretical treatments of gratitude emphasize its moral quality, much empirical research has focused on the benefits of gratitude to oneself. For example, there is convergent empirical evidence that gratitude is a positive emotion (Watkins et al., 2003; Sheldon and Lyubomirsky, 2006; König and Glück, 2014; Tong, 2014), in contrast to negative emotions like indebtedness or guilt (Müller et al., 2006; Watkins et al., 2006). Interventions that focus on gratitude increase positive affect and decrease negative affect (Emmons and McCullough, 2003; Sheldon and Lyubomirsky, 2006; Froh et al., 2008). Gratitude contributes to well-being (Wood et al., 2010), and there are positive impacts on both mental health (Lambert et al., 2012; Ng and Wong, 2013; Cheng et al., 2015; Mills et al., 2015; Van Dusen et al., 2015; Otto et al., 2016; Shao et al., 2016; Wong et al., 2016) and physical health (Jackowska et al., 2015; Redwine et al., 2016; but see Huffman et al., 2016). A recent review speculates that μ-opioids could be a potential mediator of these health effects (Henning et al., 2017). Overall, these studies establish the benefits of gratitude and distinguish it from detrimental emotions, but do not emphasize its relational and moral aspects, and they do not directly address whether gratitude increases attunement to rewards that benefit others versus the self.

### Gratitude as a Social Emotion That Involves Expression

Evidence from other domains examines the social aspects of gratitude. Expression of gratitude in personal relationships may take many forms such as returning a favor, deepening social ties to the benefactor, providing social support, or promoting social bonding (McCullough et al., 2001, 2008; Gordon et al., 2012; Algoe et al., 2013; Algoe and Way, 2014; Jia et al., 2014, 2015; Williams and Bartlett, 2015). Importantly, this urge to reciprocate can also manifest as the prosocial desire to *be* a benefactor, or “pay it forward,” to benefit the public good (DeSteno et al., 2010). A caveat is that much of this body of evidence comes from behavioral or self-report measures. Since self-reported gratitude expression is not always genuine (Baumeister and Ilko, 1995), it is important to understand whether or when charitable acts are serving to increase social status rather than being motivated by genuine care for the well-being of others (Andreoni, 1990; Eisenberg, 2006, 2014; Harbaugh et al., 2007; Decety and Cowell, 2014). Neural measures can illuminate hidden prosocial tendencies that may be obscured by self-report or behavioral measures.

Currently, there are only a few neuroimaging studies of gratitude. Instantiating gratitude in distinct ways, these studies have produced somewhat disparate conclusions on its precise neural underpinnings (Zahn et al., 2008; Fox et al., 2015; Kini et al., 2016; Yu et al., 2016) but there is ample complementary evidence that general prosociality involves reward system brain regions (Christov-Moore et al., 2014). It is reasonable to suspect that related domains, such as benevolence or prosociality, would share neural mechanisms. Recent evidence suggests that individual differences in economic choices, self-reported prosocial traits, and neural reward responses may be largely driven by a single dimension, termed “general benevolence,” which reflects a genuine altruistic concern for others (Hubbard et al., 2016). These results also indicate that prosocial motivations may increase with age in adults, but whether the neural responses underlying these propensities can be changed over a shorter time scale with an intervention in healthy young adults has not been established.

In the only study of neural change with gratitude practice, Kini et al. (2016) introduced gratitude journaling to therapy for clinical anxiety and depression with a therapy-as-usual control group. Although a pre-test scan was not acquired, an fMRI scan 3 months after treatment indicated a group difference in medial prefrontal cortex responses to gratitude ratings in the context of a giving task. Gratitude training is not unique as a prosocial intervention to change neural systems supporting altruism. In one study, Weng et al. (2013) found that individual differences in altruism were associated with compassion training-induced changes in the neural response to images of human suffering. They also found that compassion-training increased connectivity between the dorsolateral prefrontal cortex and the nucleus accumbens relative to controls in a redistribution economic task (Weng et al., 2013). These studies demonstrate that prosocial domains are trainable and provide early evidence for which brain regions may be recruited for change with training.

### The Present Study

Here, we used neuroimaging to test two hypotheses. First, consistent with the notion that gratitude motivates increased attunement toward rewards to others versus the self, we tested the prediction that self-report measures of gratitude are related to self-report measures of altruism and behavioral responses to charitable donations versus self-gains. We also tested the degree to which they may be represented as a single construct that predicts neural pure altruism, operationalized as activity in reward-related brain regions while subjects privately observe mandatory money transfers to a charity or to themselves. We chose to focus on this measure because it cannot be interpreted in terms of impure altruistic motives (e.g., signaling, warm glow, and expectation of reciprocity) because the subject is not personally responsible for the charitable transfers (Andreoni, 1990; Harbaugh et al., 2007; Hubbard et al., 2016). Also, the neural pure altruism measure is not subject to the validity threats that plague self-report measures or giving that is not private. Therefore, it provides a particularly stringent test of our individual differences hypothesis that gratitude relates to a pure form of altruism.

As a second hypothesis, we tested whether gratitude practice increases our neural measure of pure altruism, consistent with the view of gratitude as a moral and expressive emotion. More specifically, we used random-assignment and a double-blind design to assign participants to either 3 weeks of gratitude journaling or an active control journaling condition. Then we compared pre- and post-test levels of neural pure altruism between groups. Here, the neural measure allows us to test the idea that gratitude practice enhances responses that benefit others versus gains to oneself, specifically interrogating the neural system implicated in flexible determination of value (Clithero and Rangel, 2014).

## Materials and Methods

### Participants

All participants gave informed consent, all procedures were approved by the University of Oregon institutional review board and were in accord with the Declaration of Helsinki. Participants were recruited from the psychology undergraduate e-mail list at the University of Oregon and prescreened to ensure they were healthy, without MRI contraindications, right-handed, ages 18–35 years, not taking psychoactive medications, had no history of neurological or psychiatric conditions, and were willing to participate in a 3-week journaling study. All learned English as their first language and were currently living in the United States. Three participants randomly assigned to the gratitude group were lost due to attrition before the second MRI session (one informed us of an acute health issue, one informed us she was too busy to journal, and one did not complete regular journal entries and was informed she could no longer participate). None reported that they wished to withdraw due to the content of the journal entries. The final sample of participants who completed post-testing was 33 people ages 18–27 years, 16 in the Gratitude group and 17 in the Active-Neutral group (**Table 1**). We were limited by resources to this sample size, so only female participants were recruited for these experiments since gender differences in gratitude, giving behavior and neural responses to affective stimuli could increase variability (Meletti et al., 2006; Miley and Spinella, 2006; Kashdan et al., 2009; Mak et al., 2009; Stevens and Hamann, 2012). A larger sample size would more accurately estimate the magnitude of the effects (see Ingre, 2013) and allow for tests of gender differences.

**Table 1:** The 33 participants randomly assigned to Gratitude (*N* = 16) and Active-Neutral (*N* = 17) groups did not differ in age, income, or pre-test gratitude, pre-test care, or stress measures [all *T*(31) < 1.9, *p* > 0.05].

Participant summary	Mean	Range	*SD*
Age (years)	21	18–27	2
Subjective Family Income when 16-years-old (Far below average, 1; Far above average, 5)	3.4	1–5	1
Subjective Current Financial Security (Poor, 1; Average, 3; Excellent, 5)	3.2	1–5	1
Subjective Family Wealth (Few resources, 1; Ample resources, 2; Wealth, 3)	2	1–3	0.6
Pre-test Principles of Care	34	25–39	3.4
Pre-test GQ-6	37	24–42	4.7
Pre-test Stress	21	19–31	2.8

### Procedures

#### Study Overview

Participants completed a battery of online questionnaires, detailed below, the day before the first MRI scanning appointment (MRI Session 1). At Session 1 participants were shown a slide presentation with task instructions and information about the mission of a charity (a local food-bank) and then they practiced the tasks in a mock scanner. In the MRI scanner, participants completed two runs of the giving task (detailed below) followed by the anatomical scan. They also completed a task where everyday social vignettes were rated (to be reported in a separate article). Participants were blind to the aims, hypotheses, conditions, and design of the current experiment. All research-staff interacting with participants were blind to group assignment and journal content. Two cohorts were recruited, in Fall and Winter term, with testing scheduled according to the quarterly academic calendar so that post-testing at Session 2 could be completed at least 2 weeks prior to final exams. Participants were paid their task-bonus and made their donation after completion of Session 1. Bonuses ranged from $5 to $30, donations from $0 to $30, depending on the outcome of the lottery. After the second session, participants were paid the Session 2 task-bonus, made their donation, and were paid $10 per hour for testing and journaling time plus an additional $20 bonus if they had been on time.

#### Questionnaires

The main domains of interest were gratitude and altruism so our planned analysis focuses on the GQ-6 gratitude questionnaire assessing the propensity to experience gratitude in daily life (McCullough et al., 2002) and the Principles of Care, a self-report measure of altruistic moral values that is theoretically distinct from empathic concern (Wilhelm and Bekkers, 2010; Bekkers and Ottoni-Wilhelm, 2016). These two measures were acquired within in a longer battery of questionnaires.^1^

#### Intervention Procedure

##### Session 1

The day after MRI Session 1, participants were randomly assigned to one of two journaling conditions (Gratitude or Active-Neutral) and sent a link to a secure online portal (Qualtrics). Participants were instructed to write at least a 10-min journal entry every evening between dinner and bedtime for 2–3 weeks until their next MRI scanning appointment (Session 2).

##### Journaling period

We checked daily whether journal entries were completed, but did not check the contents of the entries, and we reminded participants the evening following a missed journal. On average, the Gratitude group completed 16 entries over 19 days, and the Active-Neutral group completed 18 journal entries over 19.6 days. Compliance and time between pre- and post-test did not differ between groups (**Table 2**).

**Table 2:** Groups did not differ in number of entries [*T*(31) = 1.01, *p* = 0.32] or the latency from pre-test to post-test [*T*(31) = 0.44, *p* = 0.67].

	Number of entries			Pre-Post latency (Days)		
	Mean (*SD*)	Min	Max	Mean (*SD*)	Min	Max
Gratitude	16 (1.9)	14	20	18.7 (1.4)	16	20
Active-Neutral	18 (2.8)	10	22	19.4 (2.5)	12	25

##### Journaling prompts

For the gratitude group, participants were given a standard daily prompt that was always the same (based upon Emmons and McCullough, 2003) and one of four “variety” prompts that was selected at random each day. For the active-neutral group, we designed the prompts to be engaging without a focus on gratitude (**Table 3**). Upon submission, the entry was displayed and participants indicated which prompt they had chosen and rated how they felt about their entry on a 7-point Likert scale from very unhappy to very happy. Finally, a flower name was displayed from a randomized list of common flower names (e.g., rose, daisy, and petunia) to record on a tracking sheet to aid compliance.

**Table 3:** Prompts from either the Gratitude or Active-Neutral columns, depending on group assignment, were displayed to participants each time they logged in to the online portal.

Welcome to your daily journal entry! *Some important items to remember:* You will always have two prompt choices. A general prompt that you can use every day if you like, or you may choose to write your journal entry on the second prompt given (one of four different prompts which are provided for variety). Please choose one and try to spend at least 10 min writing in your journal, responding to the prompts with as much detail as you can. Your responses are confidential. To protect your identity, please replace any names with an initial, or simply identify the relationship to you (friend, parent, sibling, roommate, etc.). Choose the daily or variety prompt below to write about today:		
	**Prompts offered to Gratitude Group**	**Prompts offered to Active-Neutral Group**

Daily (always presented)	There are many daily events in our lives, large and small that we might be thankful for. There are many people who affect our lives in a positive way. These occur in various domains, including relationships, work, school, housing, finances, health, and so forth. Think back over today or this past week and write a journal entry about what you are grateful for.	There are many daily events in our lives, large and small. They occur in various domains: relationships, work, school, housing, finances, health, and so forth. Think back over today or this past week and write a detailed journal entry of three events.
	OR	OR
Variety Prompts (one of four selected randomly per day)	Choose a person who has affected your life in a positive way who you haven’t really expressed your gratitude to. Write a letter to this person to express how you feel. If you like, you can copy your text and send it to them. What is an example of a very kind thing a stranger, or someone you don’t know well, has done for you? How did this make you feel? Describe the situation in detail. Consider how an earlier life experience has positively impacted who you are today. Choose one example and describe it in detail. Reflect on whether you have had specific advantages in life that other people may not have had. Choose one example and consider who is responsible for those advantages? How do you feel when you think about them?	Choose any person in your daily life who you don’t know very well. Write a letter to someone you DO know well describing this other person in detail. If you like you can copy your text and send it. Describe a stranger that you saw today, using as much detail as possible. Describe your typical daily routine in detail. Did anything unusual occur today? Describe a school you attended in early life with as much detail as possible.

##### Session 2

The day before Session 2, participants completed online questionnaires identical to Session 1 except without demographic questions. At Session 2, participants were informed in writing by imaging-center staff unaffiliated with the study that researchers had not read any journal entries and participants were asked not to mention their journal contents until debriefing. MRI Session 2 had the same tasks as Session 1. At debriefing, we told participants about the aims of the study and all were given information on the potential benefits of gratitude journaling.

#### Charitable Giving Task

**FIGURE 1:**
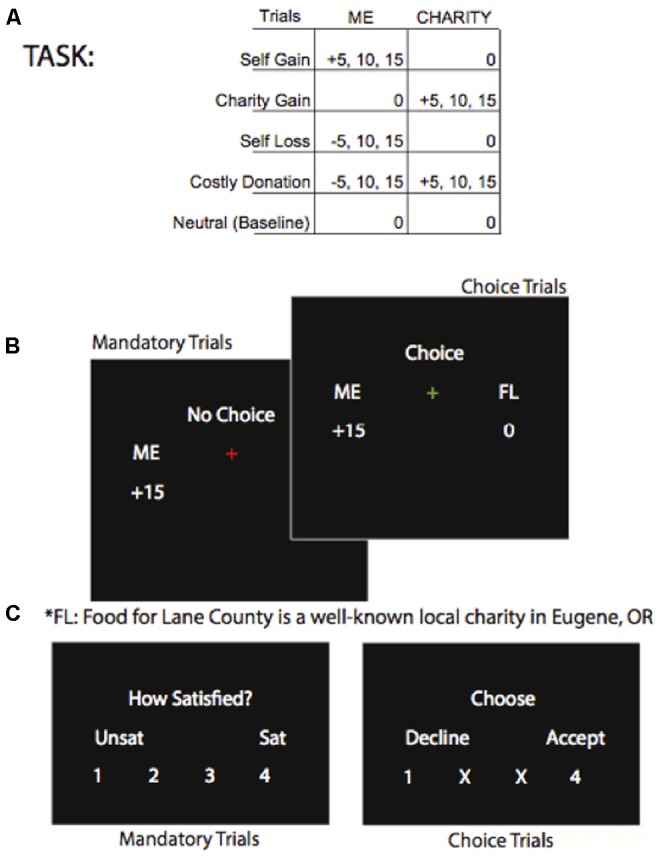
(**A**, top panel) Shows the possible trial types. (**B**, mid panel) Shows the screens for mandatory and voluntary trials showing an example of a “Self-Gain” trial type of $15 transferred to the participant and $0 transferred to the charity. (**C**, lower panel) Shows the rating screen for mandatory transfers and the rating screen for voluntary transfers.

We modified a charitable giving task based on previous work (Harbaugh et al., 2007; Hubbard et al., 2016) that allows comparison between neural responses to a charity receiving money and oneself receiving money in private transfers unobserved by experimenters. Passive transfers selected by the computer are used for the pure altruism contrast, charity-gain minus self-gain. In an instruction phase, participants read the mission of a local food-bank, were assigned a $20 bonus with the charity assigned $0, and were shown sample transfers. Transfers ranged from $0 to $15, employing various costs of giving to allow a range of responses and to reduce participant fatigue (**Figure 1A**). The proportions of mandatory and voluntary trials reflect the experimental design’s *a priori* emphasis on the neural measure of pure altruism, the mandatory passive transfers. In the task phase, 80% of trials were mandatory; a red fixation-cross indicated that participants would rate their satisfaction with the mandatory transfer on the subsequent screen (**Figure 1B**). The remaining 20% of the transfers were voluntary; a green fixation-cross indicated that the participant could either accept or reject that transfer on the subsequent screen. The participants’ selections on the final screen were highlighted as visual feedback that their button presses were detected (**Figure 1C**). Great care was taken to ensure that participants understood that experimenters were not monitoring their choices, that one mandatory and one voluntary transfer would be implemented at random, and that no deception would occur. We used a genetic algorithm (Wager and Nichols, 2003) to optimize the order of trials in this event-related design; the magnitude of gains and losses ($5, $10, $15) were assigned to trials at random following optimization.

Each run of the task, two per session, consisted of 84 trials for a total duration of approximately 10 min per run. Runs were not self-paced, and each event within each trial was jittered. Each trial began with a fixation-cross (duration 490 ms +/- 100 SD) followed by a “reveal” screen where the transfer amounts were displayed, for example “Me +0 and Charity +5” (duration 2000 ms +/- 170 SD). Next, the fixation-cross changed to red or green to indicate a voluntary or mandatory trial (duration 2500 ms +/- 150 SD). The choice or rating screen was then displayed (duration 2000 ms +/- 100 ms SD) and when a pressed key was detected, its number became green. A new trial began with the fixation-cross. After the participants exited the scanner, the lottery was implemented and they were paid in cash and given a receipt for their donation to charity. Participants had ample time to encode the transfer and make a decision prior to the display of the rating screen so response times were not analyzed.

The main dependent behavioral variable was the satisfaction ratings for mandatory transfers representing the majority of the trials. The critical tests were as follows: First, whether transfer ratings, self-reported gratitude, and self-reported altruism would be related to the neural reward system response to the pure altruism contrast at Session 1. Next, these behavioral and self-report measures were combined into aggregate variables as potential behavioral proxies of general benevolence (Hubbard et al., 2016). Third, we examined whether gratitude practice changed neural pure altruism from pre-test to post-test. Specifically, we expected that we would observe an increased response in reward-related regions, particularly the VMPFC, for charity-gains versus self-gains in line with the view that gratitude increases pure altruism.

### MRI Acquisition and Preprocessing

Imaging data were acquired using a Siemens Skyra 3.0 Tesla MRI system at the University of Oregon’s Lewis Center for Neuroimaging. Functional and anatomical brain image slices were prescribed in the mid-sagittal plane along the anterior commissure-posterior commissure (AC-PC) transverse oblique plane. For whole-brain functional images: blood oxygen-level dependent, echo-planar images (BOLD-EPI; 200 volumes per task-run) were acquired with a T2^∗^-weighted gradient echo sequence (TR = 2000 ms, TE = 30 ms, flip angle = 90°, matrix size = 64 × 64, in-plane resolution = 3.125 mm × 3.125 mm, slice thickness = 4 mm, 32 slices interleaved acquisition). For anatomical images: a high-resolution T1-weighted MPRAGE structural scan was acquired coplanar to the functional sequence (TR = 2500 ms, TE = 3.41 ms, flip angle = 7°, matrix size = 256 × 256, in-plane resolution = 1 mm × 1 mm, slice thickness = 1 mm). Foam pads minimized head movement, earplugs were worn to protect hearing, and headphones were used for communication. Stimuli were presented via the Psychophysics Toolbox within MATLAB (Brainard, 1997). A response box collected button presses from the dominant hand. We monitored alertness via a camera fixed on the right eye. After each run, researchers inquired about the participant’s comfort and alertness, and all participants had access to squeeze-ball to notify researchers of any issues requiring termination of a scan.

Images were converted from DICOM to NIfTI (Neuroimaging Informatics Technology Initiative) format using MRIconvert (mcverter version 2.0.7 build 369 ^2^) and non-brain tissue was removed using the Brain Extraction Tool implemented in FMRIB Software Library (FSL) (Smith, 2002). All subsequent processing used the Statistical Parametric Mapping toolbox (SPM version 12b; Wellcome Department of Imaging Neuroscience, London, United Kingdom) implemented in MATLAB. For each participant, functional volumes were realigned to the first image in the series. The anatomical image was registered to the realigned functional images, and reorientation parameters were manually derived and applied to all images to set the origin above and behind the anterior commissure. Anatomical images were segmented (Ashburner and Friston, 2005) and deformations fields from this transformation were used to warp functional images into standard space (MNI-152 ICBM template) at 2 mm isotropic resolution. Finally, functional images were smoothed with a 6 mm (FWHM) smoothing kernel. We used Pythagorean distance to derive four motion parameters for each volume (rotation, translation, and first derivative of rotation and translation) as regressors of no interest in the general linear model.

#### Fixed Effects Analysis for Individual Subjects

Condition effects were estimated according to a general linear model in SPM12 using a canonical hemodynamic response function, high-pass filtering (1024 s), correction for temporal autocorrelation (auto-regressive model; AR1), and a subject specific explicit mask. The Masking Toolkit in SPM12 was used to average each participant’s functional images and determine the optimal threshold for a binary exclusive mask (Ridgway et al., 2009). These individual subject masks were averaged and re-binarized to create an explicit mask for use in random-effects, group-level analyses. For measurements within regions of interest (ROI), we used Adrian Imfelds log_roi_batch v. 2.0 to extract parameter estimates^3^. The single-subject, first-level, model included five trial regressors (Charity-Gain, Self-Gain, Self-Loss, Costly Transfer by Choice, and Mandatory Costly Transfer) explicitly modeled at the onset of the transfer-type as its duration convolved with the canonical double-gamma hemodynamic response function, with the neutral condition (“Me +0 and Charity +0”) included in the implicit baseline condition. Four Pythagorean motion parameters derived from the six SPM motion parameters in the realignment procedure were added to the model as regressors of no interest. Regressors for each of the four runs (two at pre-test, two at post-test) were added to the model to allow for comparisons between Session 1 and Session 2. Responses were not explicitly modeled as nuisance regressors since all conditions contained the same responses. Planned linear contrasts were created for the main contrast of interest charity-gain versus self-gain and for each condition compared to implicit resting baseline at Session 1 and for the difference between Session 2 and Session 1. These contrasts were then entered into a random-effects group model to estimate population effects.

#### Random-Effects Analysis at the Group-Level

Analyses focused on individual differences comparisons at pre-test to test hypothesis 1, using self-report and behavioral measures as regressors of interest. To test hypothesis 2, analyses focused on the contrast indexing change in pure altruism from pre- to post-test. For these main statistical analyses, mean parameter estimates across voxels were extracted from ROIs for the pure altruism contrast (charity-gain greater than self-gain).

#### Region of Interest (ROI) Selection

We selected ROIs that are consistently implicated in subjective value: VMPFC (central, ventral, and anterior aspects; Clithero and Rangel, 2014), left and right nucleus accumbens, and left and right caudate (Harvard-Oxford probabilistic atlas thresholded at 30% and binarized in FSL; Harbaugh et al., 2007; Hubbard et al., 2016). An aggregate neural variable, the mean parameter estimate across all ROIs, was calculated as a proxy for the Neural Utility modeled in previous work (Hubbard et al., 2016). We also focused on a VMPFC aggregate in our tests of intervention effects since previous studies found associations between gratitude and regions in the medial prefrontal cortex (Fox et al., 2015; Kini et al., 2016; Yu et al., 2016).

Finally, we conducted exploratory whole-brain analyses to investigate the extent of potential coactivation with other neural systems. We modeled group differences and covariates at the second-level with whole-brain cluster extent thresholds set at family wise error rate (FWE, *p* < 0.05).

## Results and Discussion

### Hypothesis 1: Individual Differences in Gratitude and Neural Pure Altruism

First, we tested the extent to which self-reported gratitude was associated with self-report and behavioral measures of altruism at Session 1. As shown in **Table 4**, the increased levels of self-reported gratitude (GQ-6) was related to increased satisfaction ratings of mandatory costly transfers that benefited the charity [*r*(33) = 0.31, *p* < 0.05] at level similar to that of self-reported altruistic values (Principles of Care) [*r*(33) = 0.30, *p* < 0.05]. Self-reported gratitude was also robustly associated with the neural pure altruism contrast in all three VMPFC regions (*r* > 0.29, *p* < 0.05) with marginal positive associations in the nucleus accumbens (*r* > 0.25, *p* < 0.10) but not the caudate (*r* < 0.20, *p* > 0.10). Scatterplots of these relationships are provided in Supplementary Figure S1. Overall, these analyses support the hypothesis that gratitude is related to increased altruistic tendencies, and that these individual differences are supported by value-sensitive regions that have been implicated in previous studies (Harbaugh et al., 2007; Hubbard et al., 2016) but most strongly for the VMPFC.

**Table 4:** Pearson’s R values for pairwise zero-order correlations between behavioral and neural measures from independent regions of interest at pre-test for 33 participants.

	Behavioral measures			Ventromedial prefrontal cortex			Nucleus accumbens		Caudate		*Aggregate behavioral benevolence*
	Gratitude	Altruism	CostlyDon	Central	Anterior	Ventral	Left	Right	Left	Right	
Altruism	0.29^†^										
Costly donation	0.31^*^	0.30^*^									
cVMPFC	0.30^*^	0.47^*^*	0.50^*^*								
aVMPFC	0.29^*^	0.43^*^*	0.48^*^*	0.87^*^**							
vVMPFC	0.37^*^	0.46^*^*	0.61^*^**	0.91^*^**	0.82^*^**						
L Nacc	0.30^*^	0.20	0.32^*^	0.75^*^**	0.67^*^**	0.74^*^**					
R Nacc	0.25^†^	0.27^†^	0.29^*^	0.72^*^**	0.72^*^**	0.70^*^**	0.89^*^**				
L Caudate	0.19	0.13	0.23^†^	0.59^*^**	0.58^*^**	0.60^*^**	0.89^*^**	0.85^*^**			
R Caudate	0.14	0.10	0.20	0.57^*^**	0.58^*^**	0.57^*^**	0.85^*^**	0.86^*^**	0.96^*^**		
*Aggregate behavioral benevolence*	*0.73^∗∗∗^*	*0.73^∗∗∗^*	*0.74^∗∗∗^*	0.58^*^**	0.55^*^**	0.66^*^**	0.38^*^	0.37^*^	0.25^†^	0.20	
*Mean VMPFC*	0.34^*^	0.47^*^*	0.56^*^*	*0.97^∗∗∗^*	*0.94^∗∗∗^*	*0.95^∗∗∗^*	0.76^*^**	0.75^*^**	0.62^*^**	0.60^*^**	
*Neural utility, includes all ROIs*	0.30^*^	0.33^*^	0.43^*^*	*0.87^∗∗∗^*	*0.85^∗∗∗^*	*0.86^∗∗∗^*	*0.94^∗∗∗^*	*0.93^∗∗∗^*	*0.88^∗∗∗^*	*0.87^∗∗∗^*	
											0.62^∗∗∗^ 0.48^∗∗^

**FIGURE 2:**
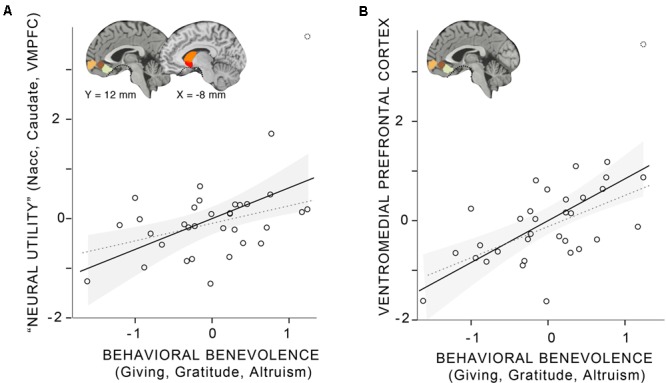
Linear relationship between the behavioral benevolence aggregate and aggregates for neural pure altruism in seven *a priori* regions of interest (ROIs) implicated in previous research (Hubbard et al., 2016). The insets show the locations of the ROIs: ventromedial prefrontal cortex (VMPFC) ROIs are those reported in Clithero and Rangel (2014). Subcortical ROIs [left and right nucleus accuments (Nacc), left and right Caudate] are Harvard Oxford atlas locations thresholded at 30%. The dashed line in ventral regions of the brain shows the limit of our sampling of the brain due to magnetic susceptibility artifacts. **(A)** The “neural utility” aggregate that includes all seven ROIs. **(B)** The aggregate across VMPFC ROI. For both panels, the shaded gray area represents the 95% confidence interval of the mean. The solid line represents the linear estimate with all participants included. The gray dashed line represents the estimate without a participant that was a potential outlier (> 3 SDs from the mean).

*Variable names in italics are aggregates. Numbers in italics with a gray background represent the item-total correlation between individual measures and the aggregate measures in which they were included. The outlined cells represent the correlations between the aggregate behavioral measures and neural measures which are also plotted in **Figure 2**. ^∗∗∗^*p* < 0.001, ^∗∗^*p* < 0.01, ^∗^*p* < 0.05, ^†^*p* < 0.10 (1-tail).*

We were also interested in whether an aggregate of the non-neural, behavioral benevolence measures was related to an aggregate of the neural measure of pure altruism, treating gratitude as part of a prosocial disposition that supports altruism, relying on a network of value-sensitive regions indexing utility. First, we created an aggregate variable incorporating self-reported gratitude, self-reported altruism, and satisfaction ratings for costly donations. After each behavioral and self-report measure was standardized and reliability was established, we averaged the scores to create a “behavioral benevolence” aggregate variable (Cronbach’s alpha = 0.83). Next we created an aggregate from the seven cortical and subcortical neural ROIs as a proxy of the neural utility measure across reward system regions reported previously by Hubbard et al. (2016) using Cronbach’s alpha (0.95) to establish reliability. The motivation for this analysis was to reduce the number of comparisons required to relate the behavior to neural activity. As shown in Supplementary Figure S1, all ROIs were robustly correlated with each other (*r* > 0.57, *p* < 0.001) with stronger associations amongst the cortical regions and themselves (*r* > 0.82, *p* < 0.001) and amongst the subcortical regions and themselves (*r* > 0.85, *p* < 0.001); these relationships are reported to demonstrate that these regions can reasonably be treated as a single entity.

As noted in the methods, medial prefrontal activity has been implicated in previous neuroimaging studies of gratitude, so separately we focused on the VMPFC ROIs. The three VMPFC ROIs were generated independently from our own data (using those from Clithero and Rangel, 2014) but since they were also highly correlated with each other, we treated them as a single construct and do not further analyze differences between the anterior, central, and ventral VMPFC ROIs. Instead, the three cortical VMPFC ROIs were standardized and averaged to create a “VMPFC utility” aggregate (Cronbach’s alpha = 0.95).

Finally, as the main analysis of interest, we examined the relationship between the behavioral and neural aggregates (**Figure 2** and **Table 4**): Behavioral benevolence was associated with the neural utility aggregate over all ROIs (*r* = 0.48, *p* < 0.01) as well as in the aggregate over VMPFC ROIs (*r* = 0.62, *p* < 0.001), providing a replication of Hubbard et al. (2016) and an extension of the general benevolence construct to self-reported gratitude and altruism. This suggests that gratitude contributes to a general prosocial disposition that supports giving, and that it is expressed in the context of pure altruism via value-sensitive brain regions, most robustly in the VMPFC.

### Hypothesis 2: Increasing Gratitude Will Increase Neural Pure Altruism

#### Changes in Self-Reported Gratitude with Gratitude Intervention

**FIGURE 3:**
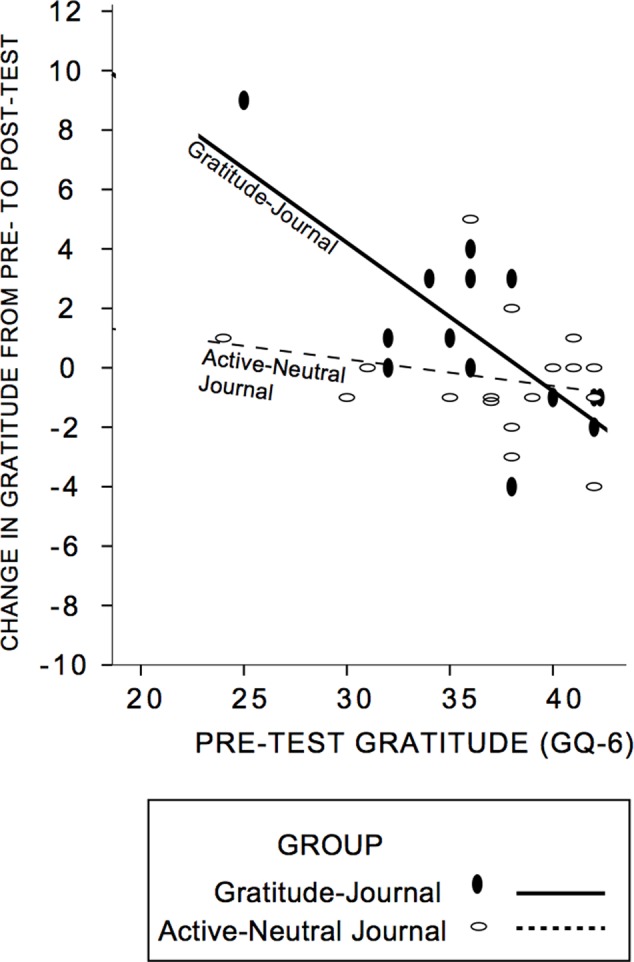
Group assignment moderated the influence of pre-test gratitude on post-test gratitude. Participants lower in pre-test gratitude who were randomly assigned to the Gratitude-Journal condition showed greater gratitude change at post-test. Solid lines and circles represent the gratitude-journal group and dashed lines and open circles represent the active-neutral control group. Supplementary Figure S2, demonstrates that estimates are not driven by the extreme values.

First, we established that our intervention was successful in increasing self-reported gratitude. We anticipated that gratitude change would be a function of pre-test gratitude scores and condition, with those lowest in gratitude changing most and conducted a hierarchical multiple regression analysis on GQ-6 change from pre- to post-test (see **Table 5** for descriptive statistics; see **Figure 3** for a scatterplot). The control group and gratitude group did not differ in pre-test levels of gratitude [*t*(30) = -0.43, *p* = 0.67]. First, pre-test gratitude and condition were included; only pre-test gratitude accounted for gratitude change and indicated that those lowest in pre-test gratitude increased gratitude most (Standardized Beta GQ-6 = -0.51, *R*^2^ = 0.30, *F*(2,29) = 6.3, *p* = 0.005]. Next, we tested whether condition (control journal group or gratitude-journal group) interacted with pre-test gratitude to impact gratitude change (Aiken and West, 1991). The interaction term accounted for an increased proportion of the variance in pre- to post-test gratitude change (Δ*R*^2^ = 0.14, Δ*F*(1,28) = 6.73, *p* = 0.015, Standardized Beta = -1.2) and the un-moderated gratitude variable was no longer significant. **Figure 3** confirms that there was an enhancing effect of the gratitude-journaling condition on people lower in pre-test gratitude, but not for the active-neutral control journaling^4^ (see also Supplementary Figure S2). The evidence is consistent with the view that the journaling condition interacted specifically with pre-test gratitude to influence change in gratitude.

**Table 5:** Pre and post-test measures in the self report, behavioral, and neural domains.

Variable	Session	Active-Neutral		Gratitude	
		Mean	*SD*	Mean	*SD*
Principles of care	Pre	34.5	3.7	33.3	3.0
	Post	33.9	4.0	33.7	2.4
Gratitude (GQ-6)	Pre	36.8	4.9	37.4	3.1
	Post	37.1	4.9	36.8	4.6
Percent give	Pre	56.7	32	58.5	28
	Post	57.2	36	57.3	30
Self-gain satisfaction	Pre	3.7	0.4	3.3	0.6
	Post	3.6	0.4	3.4	0.6
Char gain satisfaction	Pre	3.6	0.7	3.6	0.3
	Post	3.5	0.7	3.7	0.4
Costly don satisfaction	Pre	2.8	0.7	2.8	0.7
	Post	2.7	0.7	2.7	0.6
Central VMPFC	Pre	0.70	2.5	0.19	3.9
	Post	–0.43	2.8	0.77	2.7
Anterior VMPFC	Pre	1.80	2.9	0.38	4.3
	Post	–0.53	3.7	–0.10	3.5
Ventral VMPFC	Pre	0.57	1.9	0.18	3.2
	Post	–0.72	2.0	0.47	1.6
L Nacc	Pre	0.54	1.8	1.03	2.5
	Post	0.29	1.9	1.23	1.2
R Nacc	Pre	0.11	1.4	1.06	1.3
	Post	0.39	1.6	0.41	1.7
L Caudate	Pre	0.39	1.6	1.10	2.0
	Post	–0.28	1.2	0.37	1.1
R Caudate	Pre	0.38	1.3	0.73	1.5
	Post	0.09	1.2	0.23	1.2

*Neural measures represent the mean within each region of interest. VMPFC, Ventromedial prefrontal cortex. Nacc, Nucleus Accumbens. L, Left. R, Right.*

#### Changes in Behavior with Gratitude Intervention

If gratitude is related to altruism, then we would expect that a gratitude intervention would alter behavioral responses to the giving task. Since our neural measure was the pure altruism contrast (mandatory charity-gain versus mandatory self-gain), we tested for a corresponding pre- to post-test *increase* in subjective satisfaction ratings for the gratitude group versus active-neutral controls. One tailed tests were used to reflect directionality in our planned analysis given that behavioral research indicates that gratitude generally increases subjective satisfaction. Relative to controls, the gratitude group had greater satisfaction increases for both mandatory charity-gains and mandatory self-gains from pre- to post-test [**Table 5**: *T*(31) = 1.71, *p* = 0.049 1-tail; *T*(31) = 1.72, *p* < 0.048 1-tail]. We also expected *costly* donation satisfaction to increase with gratitude practice but there were no main effects of or interactions with group (*p* > 0.10). **Table 6** reports the degree of correlation between pre- and post-test for all behavioral measures and both costly donations and giving choices were highly correlated across pre- and post-test. We are unaware of any longitudinal study to determine the degree to which the evaluation of satisfaction with costly giving is changeable over time, so this null result should be interpreted cautiously. As detailed in the section below, neural measures of pure altruism were more sensitive in the current context and distinguish between gains to charity versus self.

**Table 6:** Pearson’s R values for pairwise correlations between pre- and post-test behavioral measures.

	Costly donations accepted		Ratings for mandatory trials				
	Percent		Self-Gain		Charity-Gain		Costly Donation
	Pre	Post	Pre	Post	Pre	Post	Pre
Post-percent	0.90^*^**						
Pre-self gain	–0.45^*^*	–0.43^*^*					
Post-self gain	–0.39^*^	–0.37^*^	0.75^*^**				
Pre-charity gain	0.39^*^	0.37^*^	0.04	0.01			
Post-charity gain	0.40^*^	0.40^*^	0.01	0.06	0.90^*^**		
Pre-costly donation	0.81^*^**	0.80^*^**	–0.32^*^	–0.27^†^	0.64^*^**	0.52^*^*	
Post-costly donation	0.85^*^**	0.91^*^**	–0.33^*^	–0.24^†^	0.58^*^**	0.57^*^**	0.89^∗∗∗^

*^∗∗∗^*p* < 0.001, ^∗∗^*p* < 0.01, ^∗^*p* < 0.05, ^†^*p* < 0.10 (1-tail).*

#### Changes in Brain Responses with Gratitude Intervention

##### Region of interest analysis

**FIGURE 4:**
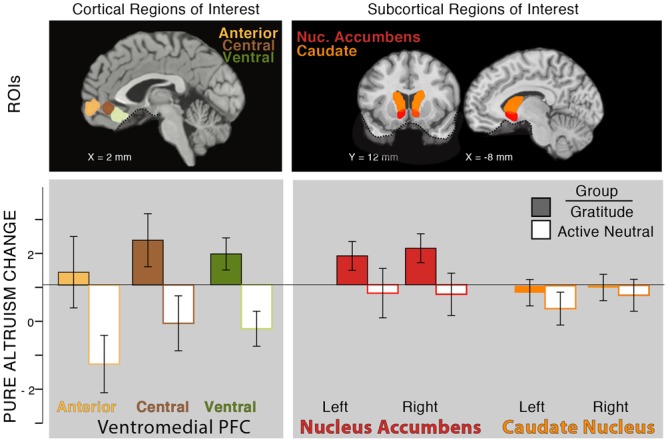
Effect of intervention on neural pure altruism in seven *a priori* ROIs implicated in previous research (Hubbard et al., 2016). The insets show the locations of the ROIs: VMPFC ROIs (left panel) are those reported in Clithero and Rangel (2014). Subcortical ROIs (right panel) are Harvard Oxford atlas locations at a threshold of 30%. The dashed line in ventral regions of the brain shows the limit of our sampling of the brain due to magnetic susceptibility artifacts. Neural pure altruism is operationalized as the contrast between the neural response to charity-gains versus self-gains for mandatory transfers of money; the *Y*-axis represents the change in neural pure altruism from pre-test to post-test. The group of participants who completed 3 weeks of gratitude journaling (colored bars) showed an overall increase in the neural pure altruism measure relative to the active-neutral journaling group (white bars) in the VMPFC.

So far, in testing our individual differences hypothesis (Hypothesis 1), we found that pre-test levels of self-reported gratitude, self-reported altruism, and satisfaction with costly transfers were associated with neural pure altruism. In testing our intervention hypothesis (Hypothesis 2), we also found that gratitude journaling increased gratitude when accounting for pre-test levels of gratitude and increased satisfaction with self-gains and charity-gains. Next, we tested whether neural pure altruism changed with gratitude practice, focusing on the VMPFC aggregate. We found that the gratitude group had a pre- to post-test increase in their neural pure altruism response relative to a decreased response for the control group [**Figure 4**: *T*(29) = 2.73, *p* < 0.05, 2-tailed].^5^

### Whole-Brain Analyses

#### Hypothesis 1, Individual Differences

**FIGURE 5:**
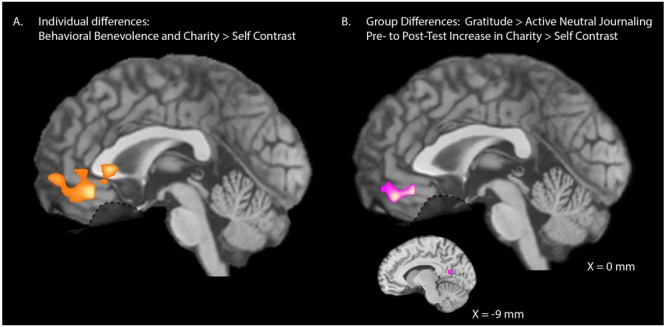
Whole-brain analyses. **(A)** Individual differences: Exploratory whole-brain analyses for the relationship between pre-test levels of behavioral benevolence and neural pure altruism. Behavioral benevolence is defined as an aggregate across self-reported gratitude, self-reported-altruism, and satisfaction with costly donations. Neural pure altruism, is defined as the contrast between mandatory charity-gains and mandatory self-gains [shown here at *T*(30) > 3.64, *p* < 0.001 2-tailed with a 53 voxel cluster correction threshold corresponding to family wise error rate (FWE) *p* < 0.05]. One VMPFC cluster survived correction [XYZ = (0, 36, –12), *K* = 173, max = 5.4, mean = 4.1]. **(B)** Intervention Effects: Exploratory whole-brain analyses for pre- to post-test change in neural pure altruism for the gratitude-journaling group versus the active-neutral journaling control group shown at a liberal threshold of *T*(29) > 2.76, *p* < 0.01 2-tail uncorrected, *K* > 20 yielding one VMPFC cluster [XYZ = (0, 45, –18), *K* = 56, max = 3.64, mean = 3.1] and one left precuneus cluster [XYZ = (–9, –60, 12), *K* = 21, max = 3.62, mean = 3.11].

Our ROIs were selected *a priori* based on previous research (Harbaugh et al., 2007; Hubbard et al., 2016) but we also explored the degree of specificity of the relationship between behavioral benevolence and neural pure altruism with whole-brain analyses to investigate whether our selected ROIs are the main regions of importance, or whether other regions may have been overlooked. We entered pre-test behavioral benevolence as a covariate at the group-level analysis. The initial height threshold of the statistical maps was *T*(30) > 3.64, *p* < 0.001, 2-tail. A cluster correction threshold of *k* > 55 voxels was applied, corresponding to FWE, *p* < 0.05. As shown in **Figure 5A**, the behavioral benevolence aggregate was positively associated with the pure altruism contrast in a single region of the VMPFC [XYZ = (0, 36, -12), *K* = 173, max = 5.4, mean = 4.1].

#### Hypothesis 2, Journal Condition Effects

We also explored the specificity of the change from pre- to post-test with gratitude journaling for the pure altruism contrast. At an initial threshold of *p* < 0.001, no voxels survived cluster correction at FWE < 0.05. At a liberal threshold (uncorrected *p* < 0.01, *k* > 20) a cluster in the VMPFC was implicated [**Figure 5B**: XYZ = (0, 45, -18), *k* = 59] along with a left precuneus cluster [XYZ = (-9, -60, 12), *k* = 23] a region involved in subjective value (Clithero and Rangel, 2014). This evidence suggests that the gratitude intervention operated primarily on cortical reward networks associated with context dependent value.

## Conclusion

Previous research on gratitude has emphasized either its positive effects on the self or its relational nature, in terms of expressing gratitude to a benefactor or paying a benefit forward to others. We tested the prediction that gratitude fosters attunement toward rewards to others in two ways: First we took an individual differences correlational perspective to test whether self-reported gratitude and altruism, and satisfaction with charitable donations are associated with neural measures of pure altruism (the increased signal in reward-related regions for mandatory gains to a charity compared to mandatory gains to oneself). Second, we took an experimental approach to test whether 3-weeks of gratitude practice through journaling would change neural pure altruism. Our results from both approaches support the view that gratitude is associated with increased altruism. In other words, gratitude biases the brain’s reward system toward rewards for others versus oneself.

Using an established task (Harbaugh et al., 2007; Hubbard et al., 2016), we found that trait levels of gratitude were associated with neural pure altruism (greater neural response to charity-gains than self-gains) in reward system brain regions, particularly the VMPFC. We also replicated and extended prior work by demonstrating that an aggregate behavioral measure of benevolence (gratitude, altruism, and satisfaction) was related to neural measures of pure altruism (Hubbard et al., 2016). Furthermore, in this first neuroimaging study of longitudinal change in altruism with a gratitude practice, we found that neural measures of pure altruism increased for the gratitude group relative to the active-neutral control group in cortical reward system regions centered on the VMPFC. This evidence positions the VMPFC as an important brain region supporting the altruistic change that follows from gratitude practice.

In regards to our individual differences approach, there are few neuroimaging studies relating self-reported propensity toward gratitude to neural activity, and each takes a different approach to operationalizing gratitude and investigating its neural underpinnings (Zahn et al., 2008; Fox et al., 2015; Kini et al., 2016; Yu et al., 2016). One study of patients seeking treatment for anxiety and depression reported an association between a self-report measure of gratitude (GAC3, a three item adjectives scale; McCullough et al., 2002) and medial prefrontal activity related to trials where participants gave more in a reciprocal donation task, but its locus is more caudal than our VMPFC ROI and our locus in the whole-brain analysis. In this same study the GQ-6 measure of gratitude was only associated with somatomotor activity related to trials where participants expressed a greater desire to help (Kini et al., 2016). Another study utilizing a whole-brain approach found that the GQ-6 was related only to a posterior cingulate and precuneus region in a paradigm involving shared pain (Yu et al., 2016). The inconsistencies across studies are likely due to different means of operationalizing and modeling both trait and experienced gratitude. Future research is needed to clarify the degree to which different operationalizations of gratitude affect the neural systems engaged in a task and how this affects these trait-level associations. In the current study, we took a targeted approach to understand how trait gratitude relates to the construct of pure altruism, which avoids impure motivations such as the warm glow with voluntary giving, expectations of reciprocity, or social benefits such as signaling generosity. This is not to say that there are not other contexts in which gratitude may be expressed differently, but serves to clarify the role of gratitude within a specific theoretical conception of altruism.

An important question is the degree to which the findings are specific to the gratitude manipulation. Other factors in the journal prompts could elicit changes in more domains than just gratitude, such as overall expressiveness, positive affect, or empathy. Future work could analyze the content of the journals or introduce other self-report measures to clarify the degree to which gratitude is a special path to increased altruism. This could also clarify the extent to which it shares features with other domains that can increase altruism such as mindfulness or compassion training (e.g., Weng et al., 2013). Overall, there seems to be converging evidence from multiple domains that indicates a relationship between gratitude and altruism (e.g., Roberts, 2015; Tsang and Martin, 2017). Our study is a piece of evidence that grateful generosity need not be reciprocal, since our manipulation involved a group charity, rather than a specific person as a beneficiary, and our key measure was the pure altruism contrast.

Although neural research on gratitude is still in its infancy and discrepancies need to be resolved, we are reassured by some consistency across neuroimaging studies. The current work highlights the importance of the VMPFC for both trait-level individual differences measures in self-reported gratitude and altruism as well as change with gratitude practice. A task-related gratitude contrast in the study by Yu et al. (2016) also highlights the VMPFC and a nearby pregenual anterior cingulate region showed group differences at post-test (pre-test measures were not collected) for a therapy group receiving a gratitude component relative to the therapy-as-usual group in patients with anxiety and depression (Kini et al., 2016). Thus the VMPFC is a promising focus for future work on gratitude and other prosocial moral emotions. Individual differences and lifespan research will also provide important insights into broad or specific domains to target for intervention, neural systems that are most receptive to training, as well as which individuals might benefit most from training. Despite these unanswered questions, these are the first randomized and controlled data in healthy participants to show pre-test to post-test changes in BOLD signal as a result of gratitude training following a relatively brief training period.

Our findings were also largely consistent with those from a recent study using the same charity-gain versus self-gain contrast as a measure of pure altruism. This study found that a prosocial disposition, giving choices, and increasing age all were associated with increased signal in the same ROIs we used in the current study, and most prominently in the VMPFC (Hubbard et al., 2016). Although our aggregate measures of self-reported gratitude and altruistic values are different than the self-report measures of general benevolence described by Hubbard et al. (2016), it is reasonable to suspect that gratitude and values that emphasize altruism and care for others reflect aspects of general benevolence. Future work with larger samples and more diverse measures is needed to clarify the degree to which these separable contributions to general benevolence may explain distinct rather than unitary aspects of altruistic motivations or behavior.

An important advance in the current study is our direct test of the hypothesis that gratitude training can increase neural pure altruism, as indexed via reward-system responses. Although previous behavioral empirical work emphasizes the role of gratitude in giving (e.g., DeSteno et al., 2010) a view of gratitude persists, particularly in the popular press, focusing on potential gains-to-self (see Ehrenreich, 2015, for a critique). Conversely, a moral and expressive view of gratitude, more in line with current theoretical approaches, predicts that practice would increase the perceived value of rewards to others. Because we acquired fMRI data before and after gratitude training (in contrast to Kini et al., 2016), we were able to directly demonstrate that the VMPFC changes its responses to rewards for others with gratitude practice.

In summary, we establish that grateful people show stronger neural signatures of pure altruism in reward system regions. Furthermore, after 3 weeks of journaling, participants who were randomly assigned to the gratitude condition engaged the VMPFC more strongly for altruistic transfers of money than participants in a similar, but neutral, journaling condition. This indicates that the VMPFC, a context-dependent value-sensitive region, may underlie altruistic change following a gratitude practice. More broadly, we demonstrate that neural measures that reflect a genuine concern for others change after an intervention that targets a prosocial and moral emotion.

## Author Contributions

CK initiated the project, designed the experiment, performed and supervised data collection, performed analyses, and wrote the manuscript. WM assisted with design and implementation, performed analysis, and contributed to the manuscript. UM consulted on design, analysis, and interpretation of results, and contributed to the manuscript.

## Conflict of Interest Statement

The authors declare that the research was conducted in the absence of any commercial or financial relationships that could be construed as a potential conflict of interest.

## Footnotes

1 Additional questionnaires were acquired for future exploratory analyses and to ensure that participants would be less likely to be aware of the specific study aims. Questionnaires were presented in randomized order, except for demographic questions, which were presented first and only at Session 1. Demographic Questionnaires: Age, Gender, whether English was their first language, whether they were currently living in United States, their subjective family income at age 16 relative to other American families, their current subjective financial security, their family wealth, socioeconomic status of origin (Hollingshead questionnaire). Other Questionnaires: Tolerance of Financial Risk, Principles of Care, Gratitude (GQ-6), Life Satisfaction, Subjective Happiness, Mindfulness (Five-Factor Mindfulness Inventory), Life-Stress Events, Short Big-5 (BFI-10), Assessment of Engagement, Political Identity, Religiosity/Spirituality, Narcissism (NPI-16), and a paragraph about current stressors.

2 http://lcni.uoregon.edu/∼jolinda/MRIConvert

3 http://www.fil.ion.ucl.ac.uk/spm/ext/

4 We also excluded two participants lowest in gratitude and the moderation model was still significant, indicating that the estimates are not driven by extreme values [see **Supplementary Figure S1**; Standardized Beta GQ-6 = -0.41, *R*^2^ = 0.17, *F*(2,28) = 5.71, *p* = 0.024]. As an exploratory step, we ran a moderation analysis on the behavioral benevolence aggregate but this did not reach significance: Pre-test behavioral benevolence on gratitude change [Standardized β = -0.28, *R*^2^ = 0.06, *F*(2,29) = 1.94, *p* > 0.10] and did not interact with condition to influence gratitude change [Δ*R*^2^ = 0.09, Δ*F*(1,28) = 3.1, *p* > 0.05, Standardized β = -0.90].

5 As an exploratory step, we tested whether gratitude practice changed the neural utility aggregate across all seven ROIs but this did not reach statistical significance [*T*(29) = 2.00, *p* = 0.056]. Instead the effect was centered on the VMPFC as shown in the main analysis and the whole-brain analysis.
