# Supplementary material for: The Cultivation of Pure Altruism via Gratitude: A Functional MRI Study of Change with Gratitude Practice
Source: Front Hum Neurosci. 2017 Dec 12;11:599. doi: 10.3389/fnhum.2017.00599 (PMC5770643; doi:10.3389/fnhum.2017.00599)
Supplement: Supplementary file 1 [file Image_1.PDF]

SUPPLEMENTARY FIGURE S1

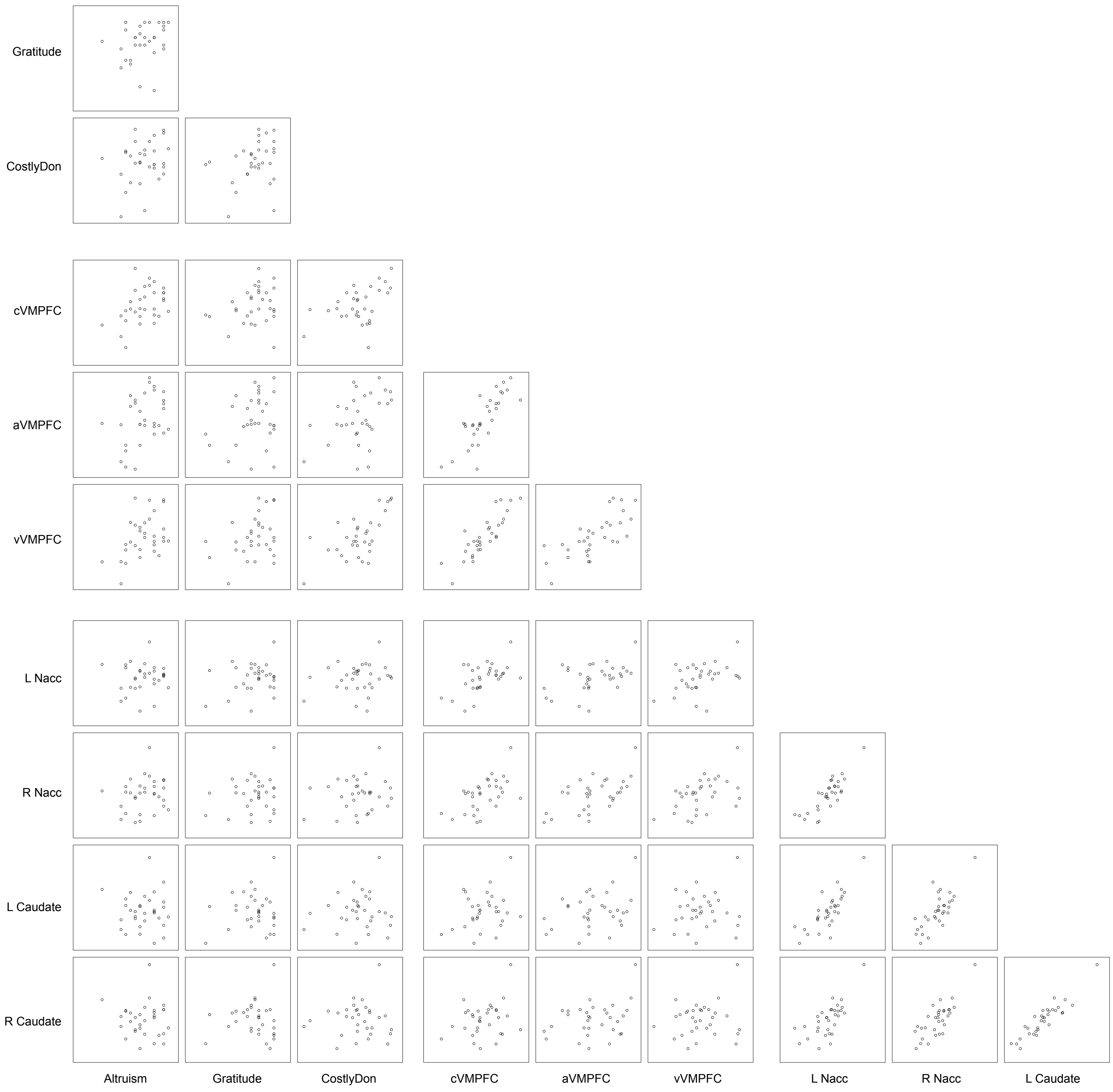

Supplementary Figure S1

Scatterplots of the relationships between behavioral measures (altruism, gratitude, and satisfaction ratings for costly donations), ventromedial prefrontal cortex ROIs (central, anterior, and ventral), and subcortical ROIs (left and right nucleus accumbens, and left and right caudate). The Pearson's R values are reported in Table 4.
