# Supplementary material for: The Cultivation of Pure Altruism via Gratitude: A Functional MRI Study of Change with Gratitude Practice
Source: Front Hum Neurosci. 2017 Dec 12;11:599. doi: 10.3389/fnhum.2017.00599 (PMC5770643; doi:10.3389/fnhum.2017.00599)
Supplement: Supplementary file 2 [file Image_2.PDF]

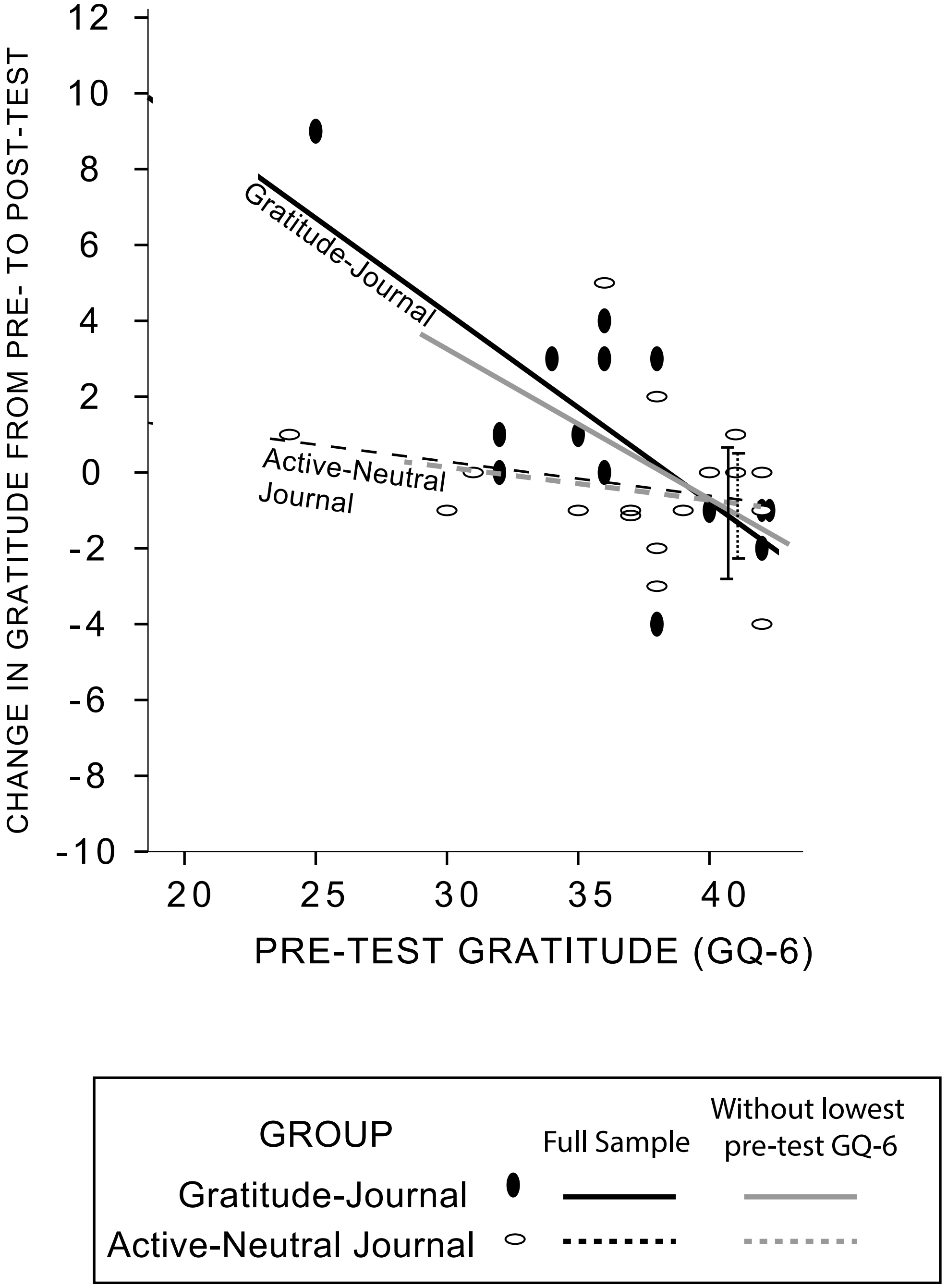

Supplementary Figure S2

Group assignment moderated the influence of pre-test gratitude on post-test gratitude. Participants lower in pre-test gratitude who were randomly assigned to the Gratitude-Journal condition showed greater gratitude change at post-test. Solid lines and circles represent the gratitude journal group and dashed lines and open circles represent the active-neutral control group. 95% confidence intervals are plotted at higher scores to show that groups did not differ in gratitude change at higher pretest scores. The gray lines are the linear estimates with the two participants lowest in gratitude (one from each group) excluded to demonstrate that estimates are not driven by the extreme values.
